# Whole-genome sequence data and analysis of *Saccharibacillus* sp. ATSA2 isolated from Kimchi cabbage seeds

**DOI:** 10.1016/j.dib.2019.104465

**Published:** 2019-08-31

**Authors:** Lingmin Jiang, Chan Ju Lim, Jae Cheol Jeong, Cha Young Kim, Dae-Hyuk Kim, Suk Weon Kim, Jiyoung Lee

**Affiliations:** a Korean Collection for Type Cultures (KCTC), Biological Resource Center, Korea Research Institute of Bioscience and Biotechnology (KRIBB), Jeongeup, 56212, Republic of Korea; b Department of Bioactive Materials, Chonbuk National University, Jeonju, 54896, Republic of Korea; c Asia Seed Co., Research Institute of Biotechnology Breeding, Icheon, 17414, Republic of Korea

**Keywords:** *Saccharibacillus* sp. ATSA2, Complete genome sequence, Kimchi cabbage seeds, Rapid annotation subsystem technology (RAST), RAST, rapid annotations subsystems technology, HGAP, hierarchical genome assembly process, SMRT, single-molecule real-time, rRNA, ribosomal RNA, tRNA, transfer RNA, ncRNA, non-coding RNA, PHO, phosphate

## Abstract

*Saccharibacillus* sp. ATSA2 was isolated from Kimchi cabbage seeds grown in Gyeongbuk province in the Republic of Korea. Whole-genome sequencing of *Saccharibacillus* sp. ATSA2 was performed using the PacBio RSII and Illumina HiSeq platforms, and it features a 5,619,468 bp circular chromosome with 58.4% G + C content. The genome includes 4543 protein-coding genes, 104 RNA genes (70 transfer RNA genes, 30 ribosomal RNA genes, and 4 non-coding RNA), and 73 pseudogenes. Multiple gene clusters associated with stress responses, nitrogen and phosphorus metabolism, and plant hormone biosynthesis were annotated in the genome. The genome information will provide fundamental knowledge of this organism as well as insight for understanding microbial activity in the agricultural application. The whole-genome sequence of *Saccharibacillus* sp. ATSA2 is available at GenBank/EMBL/DDBJ under accession number CP041217.

**Table:** Specifications Table

Subject	Biology
Specific subject area	Microbiology and genomics
Type of data	Complete genome sequence data in FASTA format, figure and image
How data were acquired	Genome sequencing platform: PacBio RSII; Illumina HiSeq Genome annotation: NCBI Prokaryotic Genome Annotation Pipeline (PGAP); Rapid Annotations Subsystems Technology (RAST)
Data format	Analyzed and assembled genome sequences
Parameters for data collection	Genomic DNA was extracted from a pure culture of *Saccharibacillus* sp. ATSA2
Description of data collection	Whole-genome sequencing, assembly, and annotation
Data source location	Strain ATSA2 was isolated from Kimchi cabbage seeds grown in Gyeongbuk province, Republic of Korea (36° 39′ 27″ N/128° 27′ 19″ E)
Data accessibility	The complete genome sequence of *Saccharibacillus* sp. ATSA2 has been deposited to GenBank under accession number CP041217 (https://www.ncbi.nlm.nih.gov/nuccore/CP041217.1), BioProject number PRJNA544163; BioSample number: SAMN11812191.
Related research article	*Saccharibacillus brassicae*sp. nov*.,*an endophytic bacterium isolated from Kimchi cabbage seeds (*Brassica rapa*subsp. pekinensis); Journal of microbiology, “under review”

**Table:** 

**Value of the data** • The complete genome sequence of *Saccharibacillus* sp. provides fundamental knowledge of this organism and insight for understanding its microbial activity and biotechnological application in agriculture. • The complete genome sequence data are useful for the comparative genomic study of *Saccharibacillus* species and can be used by other researchers studying *Saccharibacillus* species to obtain bioinformation or for microbiology genome analysis. • The complete genome sequence data will be useful for studying the stress responses of the *Saccharibacillus* species, and exploring its useful metabolism.

## 1 Data

The genus *Saccharibacillus* belongs to the family *Paenibacillaceae* within the phylum *Firmicutes*, as established by Rivas [1]. There are 5 species with validly published names (http://www.bacterio.net/saccharibacillus.html) as of August 2019. The species were isolated from different environmental niches, including desert soil [2], [3], sugar cane [1], lead-cadmium tailing soil [4], and endophyte of cotton [5]. Strain ATSA2 was isolated from surface-sterilized Kimchi cabbage seeds grown in the Gyeongbuk province of Korea, regarded as a novel species of *Saccharibacillus* based on its 16S sequence highest similarity to *S. deserti* WLG055^T^ (98.1%), which is below the proposed novel species recognition threshold of 98.6% [6]. Because the type strain *S. deserti* strain WLG055^T^ genome has not been performed by the other researchers, it is impossible to obtain the unequivocal position of the novel strain ATSA2. Complete genome sequencing of strain ATSA2 was performed in order to better understand this organism.

Whole-genome sequencing was performed using the PacBio RSII (Pacific Biosciences Inc.) and the Illumina HiSeq X-Ten (Macrogen Inc.) platforms. A total of 136,625 sub-read (N50 value was 12,509 bp) and 1,233,875,839 sub-read base pairs with coverage of 272× were generated, and these sub-reads were assembled using the RS hierarchical genome assembly process (HGAP) (v3.0) [7] and single-molecule real-time (SMRT) Portal (v2.3) [8] *de novo* assembler. The genome was annotated using the PGAP with best-placed reference protein set GeneMarkS 2 (v4.7) [9] and the RAST server (http://rast.nmpdr.org/) [10].

**Fig. 1:**
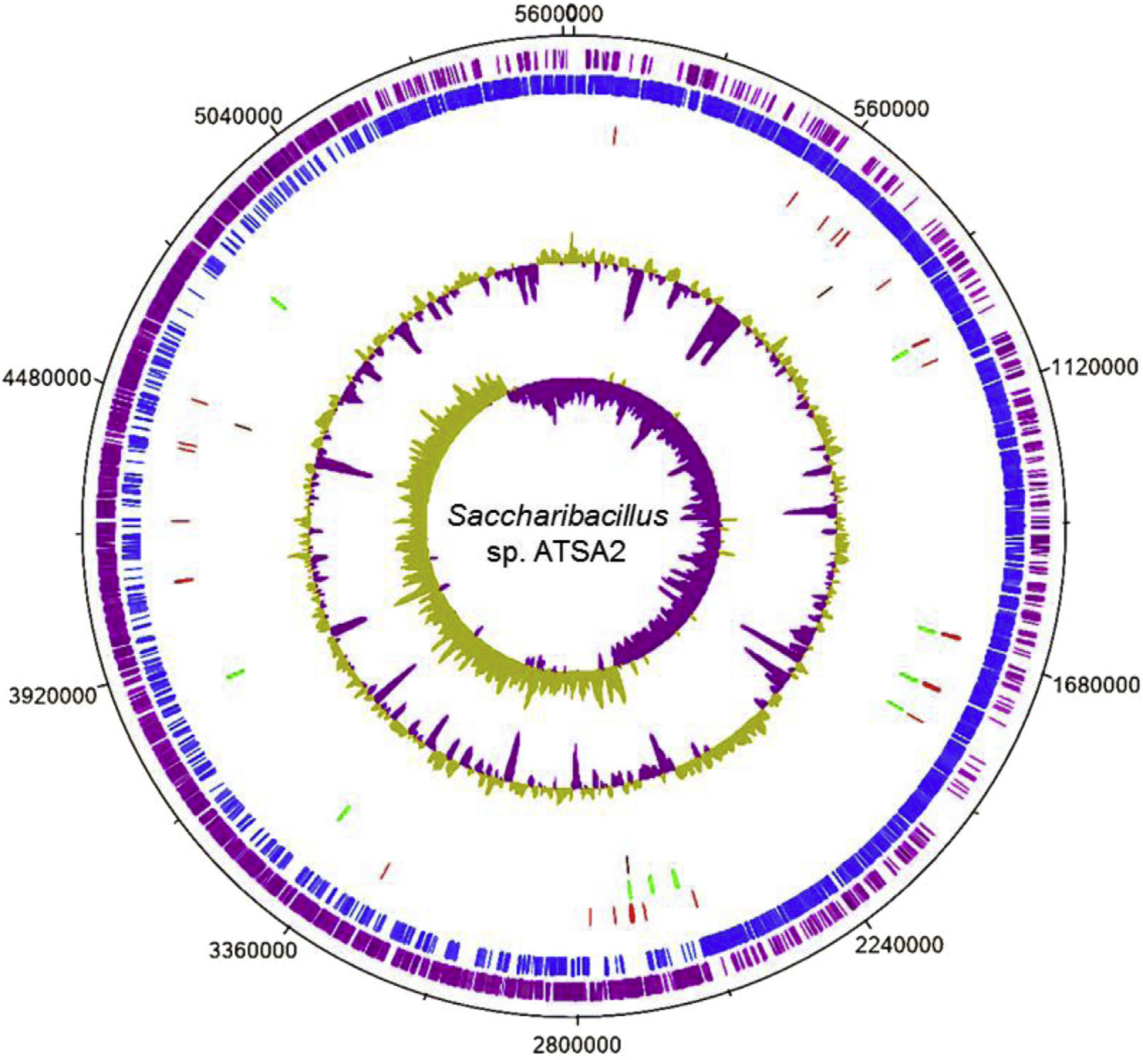
Graphical circular map of the genome of *Saccharibacillus* sp. ATSA2. The rings from the outside toward the center show the following: track 1, genome size; track 2, forward CDSs (purple); track 3, reverse CDSs (blue); track 4, tRNA (red); track 5, rRNA (green); track 6 (black), tracks 7, ncRNA, %GC plot; tracks 8, GC skew [(G − C)/(G + C)].

The complete genome of *Saccharibacillus* sp. ATSA2 was composed of a 5,619,468 bp circular chromosome with 58.4% G + C content. It was determined that the genome contains 4543 coding sequences (CDSs), 30 rRNAs (10 copies of 5S, 16S, and 23S, respectively), 70 tRNAs, 4 ncRNAs, and 73 pseudogenes. The genome features of *Saccharibacillus* sp. ATSA2 are summarized in Fig. 1.

**Fig. 2:**
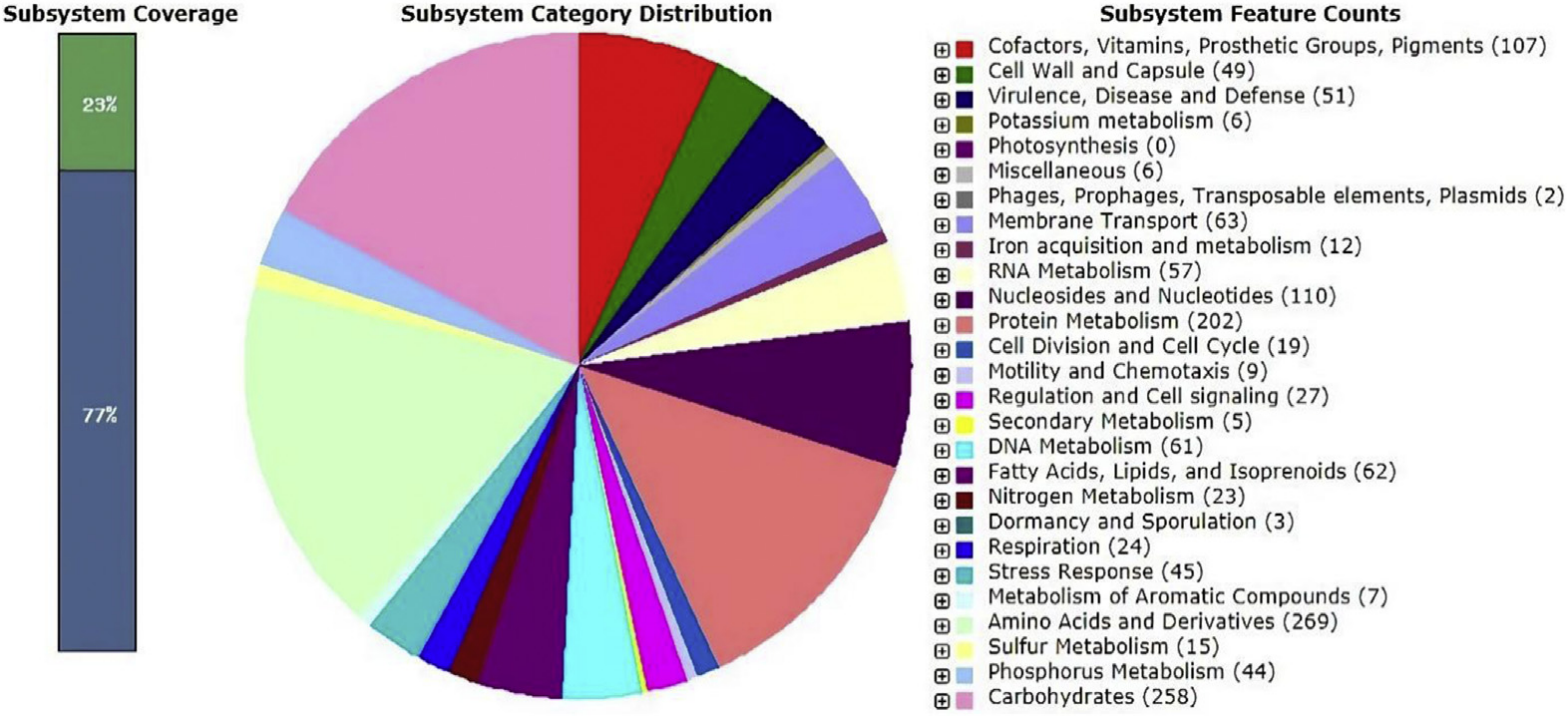
An overview of the subsystem categories assigned to the genome of *Saccharibacillus* sp. ATSA2. The whole-genome sequence of the strain ATSA2 was annotated using the RAST server.

An analysis obtained from the RAST server revealed that the *Saccharibacillus* sp. ATSA2 genome contains 5018 coding sequence and 296 subsystems (Fig. 2). The most represented subsystem features are amino acids and derivatives (269), carbohydrates (258), protein metabolism (202), cofactors, vitamins, prosthetic group, and pigments (107). RAST also identified 45 genes clusters for stress response (osmotic stress, 6; oxidative stress, 20; detoxification, 2; no subcategory, 15; and periplasmic stress response, 2), 23 nitrogen metabolism (subcategory, 19 and denitrification, 4), 5 secondary metabolism (auxin biosynthesis, 5), and 44 phosphorus metabolism (phosphate metabolism, 28; high affinity phosphate transporter and control of PHO regulon, 14; and polyphosphate, 2) in the whole-genome annotation. These will provide basic understanding and facilitate future research on this bacterium.

## 2 Experimental design, materials, and methods

Genomic DNA was extracted from *Saccharibacillus* sp. ATSA2 cell pellets using a genomic DNA purification kit (MGmed, Republic of Korea). The *Saccharibacillus* sp. ATSA2 genome was sequenced at Macrogen (Seoul, Republic of Korea) using both the Illumina HiSeq X-Ten (150 bp paired-end sequencing) and PacBio RSII (Pacific Biosciences, USA) platforms. Library preparation for Illumina and PacBio sequencing was performed using the TruSeq DNA sample prep kit for Illumina (NE, USA) and the PacBio DNA template prep kit (Pacific Biosciences, USA), respectively, according to the manufacturers' instructions. The library insert sizes were 350 bp for Illumina sequencing and 20 kb for PacBio RS SMRT sequencing. Ultimately, 10,138,164 paired-end reads were generated by Illumina sequencing, and 136,625 long reads were generated by PacBio sequencing. Trimmed reads generated by Trimmomatic 0.32 software were used for *de novo* assembly based on the HGAP3 using SMRT portal (v2.3). To obtain a high-quality sequence, error correction of the assembled contig was performed by hybrid assembly using Illumina raw sequence data. This resulted in one contig representing a complete chromosome sequence (N50, 12,509 bp; final coverage, 272×). The annotation was carried out with the NCBI PGAP through the NCBI Genome submission portal (Genome Submit at http://ncbi.nlm.nih.gov). Chromosome topology was drawn using DNAPlotter [11]. Gene prediction was accomplished on the Rapid Annotation using the Subsystem Technology SEED viewer (RAST; http://rast.nmpdr.org/).
